# Infection of liver sinusoidal endothelial cells with Muromegalovirus muridbeta1 involves binding to neuropilin-1 and is dynamin-dependent

**DOI:** 10.3389/fcimb.2023.1249894

**Published:** 2023-11-09

**Authors:** Ingelin Kyrrestad, Anett Kristin Larsen, Javier Sánchez Romano, Jaione Simón-Santamaría, Ruomei Li, Karen Kristine Sørensen

**Affiliations:** Department of Medical Biology, Faculty of Health Sciences, UiT - The Arctic University of Norway, Tromsø, Norway

**Keywords:** LSEC, dynasore, MitMAB, endocytosis, cytomegalovirus, MuHV-1, CMV, neuropilin-1

## Abstract

Liver sinusoidal endothelial cells (LSEC) are scavenger cells with a remarkably high capacity for clearance of several blood-borne macromolecules and nanoparticles, including some viruses. Endocytosis in LSEC is mainly via the clathrin-coated pit mediated route, which is dynamin-dependent. LSEC can also be a site of infection and latency of betaherpesvirus, but mode of virus entry into these cells has not yet been described. In this study we have investigated the role of dynamin in the early stage of muromegalovirus muridbeta1 (MuHV-1, murid betaherpesvirus 1, murine cytomegalovirus) infection in mouse LSECs. LSEC cultures were freshly prepared from C57Bl/6JRj mouse liver. We first examined dose- and time-dependent effects of two dynamin-inhibitors, dynasore and MitMAB, on cell viability, morphology, and endocytosis of model ligands via different LSEC scavenger receptors to establish a protocol for dynamin-inhibition studies in these primary cells. LSECs were challenged with MuHV-1 (MOI 0.2) ± dynamin inhibitors for 1h, then without inhibitors and virus for 11h, and nuclear expression of MuHV-1 immediate early antigen (IE1) measured by immune fluorescence. MuHV-1 efficiently infected LSECs *in vitro*. Infection was significantly and independently inhibited by dynasore and MitMAB, which block dynamin function via different mechanisms, suggesting that initial steps of MuHV-1 infection is dynamin-dependent in LSECs. Infection was also reduced in the presence of monensin which inhibits acidification of endosomes. Furthermore, competitive binding studies with a neuropilin-1 antibody blocked LSEC infection. This suggests that MuHV-1 infection in mouse LSECs involves virus binding to neuropilin-1 and occurs via endocytosis.

## Introduction

Betaherpesviruses are enveloped DNA viruses (size 150-200 nm) that can infect a variety of organs and cell types and establish life-long latency (i.e., lifelong carriage of virus without symptoms). Endothelial cells play an important role in disease and latency of human and mouse betaherpesviruses, also known as cytomegaloviruses (Sinzger et al., 1995; Jarvis and Nelson, 2002; Krmpotic et al., 2003; Jarvis and Nelson, 2007; Reddehase and Lemmermann, 2019; Munks et al., 2023). Infection is species specific, which excludes experimental infection of research animals with cytomegalovirus humanbeta5 (HHV-5, aka human cytomegalovirus) (Brizić et al., 2018). Muromegalovirus muridbeta1 (MuHV-1, aka murine cytomegalovirus) has therefore been extensively used as a model for human HHV-5 infection in mice, which has generated important knowledge about viral pathogenesis and the discovery of immune-evasive genes (Brizić et al., 2018; Fisher and Lloyd, 2021).

Cellular entry of human HHV-5 has been well studied in several cell types (Vanarsdall and Johnson, 2012), and *in vitro* studies have shown that HHV-5 enter endothelial cells (transformed human umbilical vein endothelial cells) via endocytosis (Ryckman et al., 2006; Martinez-Martin et al., 2018). In a mouse model, neuropilin-1 (Nrp1) has recently been reported as a potential entry receptor for MuHV-1 in endothelial cells, fibroblasts, and macrophages. However, there is still a shortage of information about the entry pathways utilized by MuHV-1 (Lane et al., 2020). As MuHV-1 is used as an *in vivo* model for human HHV-5, more knowledge about the initial steps of infection in target cell types, like endothelial cells, is needed in the mouse model. Endothelial cells are heterogenous across tissue vascular beds, reflecting organ-specific functions, but also vary in their structure and phenotype between vessel types within the organ (Jarvis and Nelson, 2007). The infection pathways may thus vary between endothelial cell types.

The endothelial cells that line the sinusoids in the liver, i.e. the liver sinusoidal endothelial cells (LSECs) make up a specialized, fenestrated endothelium with an extraordinarily high clearance capacity for blood-borne waste macromolecules, and various nanoparticles (< 100-200 nm), including some viruses and virus-like particles (Ganesan et al., 2011; Simon-Santamaria et al., 2014; Mates et al., 2017), thus contributing to the maintenance of homeostasis (Sørensen et al., 2012; Bhandari et al., 2021). The LSECs have a well-developed endocytic apparatus and express high levels of scavenger receptors, C-type lectins, and other receptors tailored to bind and internalize an array of endogenous and exogenous ligands, which are then efficiently degraded in the cells (Gramberg et al., 2005; Sørensen et al., 2012; Shetty et al., 2018; Bhandari et al., 2020; Pandey et al., 2020; Bhandari et al., 2021). Together with the liver macrophages (i.e. Kupffer cells), LSECs constitute the most powerful scavenger cell system in the body (Sørensen et al., 2012). However, in contrast to macrophages, LSECs are non-phagocytic cells, and uptake of ligands via LSEC signature receptors occur via clathrin-coated pit-mediated endocytosis (Eskild et al., 1989; Esbach et al., 1994; Hellevik et al., 1998; Kjeken et al., 2001; Hansen et al., 2005; Mousavi et al., 2007; Bhandari et al., 2021). Many viruses enter cells via clathrin-mediated endocytosis. There are, however, rather few viruses that are reported to cause infection in LSECs (Breiner et al., 2001; Peyrefitte et al., 2006; Seckert et al., 2009; Zellweger et al., 2010; Kondo et al., 2021), and the hypothesis has been presented that LSECs due to their scavenging activity and effective catabolism of endocytosed ligands have a role in the body's antiviral defense by eliminating circulating viruses (Seternes et al., 2002; Ganesan et al., 2011; Sørensen et al., 2012). The high rate of endocytosis may also be a double-edged sword and facilitate uptake of certain pathogenic viruses.

Among the few viruses that are known to infect LSECs are HHV-5 (in human) and MuHV-1 (in mouse), and LSECs are an important site of persistence of these viruses (Sacher et al., 2008; Seckert et al., 2009; Bosch et al., 2011; Bruns et al., 2015). However, the early stages of infection in LSECs have not been characterized. We hypothesized that LSECs, due to their dominating clathrin-mediated endocytosis, are likely to be infected with MuHV-1 via this route, which is a dynamin-dependent route of entry (Cheng et al., 2021). Dynamin is a 96 kDa GTPase that mediates the scission of the forming vesicle in distinct endocytic pathways, including clathrin-mediated endocytosis, and fast endophilin A-mediated endocytosis (Mettlen et al., 2009).

One aim of the present study was to examine the role of dynamin in MuHV-1 early infection in primary mouse LSECs. To this end we used two dynamin-inhibitors that operate via different mechanisms, dynasore, and MitMAB (myristyl trimethyl ammonium bromide). Dynasore is a well-studied small-molecule noncompetitive inhibitor of dynamin that blocks dynamin scission (Macia et al., 2006; Preta et al., 2015), and has been extensively used in studies of entry and infection of different viruses in cell culture systems (Abban et al., 2008; Huang et al., 2011; Mulherkar et al., 2011; Rahn et al., 2011; Lee et al., 2013; Carro et al., 2018; Bayati et al., 2021). There are, however, few studies on the use of dynasore in primary cells (Barrias et al., 2010; Rahn et al., 2011; Mues et al., 2015). MitMAB is another GTPase inhibitor that targets the interaction of dynamin with phospholipids and thereby blocks dynamin recruitment to membranes (Hill et al., 2004). Apart from a recent study where dynasore was used to inhibit VEGF-receptor-mediated endocytosis in rat LSECs in suspension (Mousavi et al., 2019), the effect of dynasore, or MitMAB on LSECs has not been reported. We therefore first investigated dose- and time-dependent effects of dynasore and MitMAB on LSEC viability, ultrastructure, and endocytosis of model ligands operating via LSEC signature receptors, to establish the protocol for the use of the inhibitors in MuHV-1 infection studies. Since LSECs are well known to change their phenotype in culture and rapidly lose their scavenging capacity and fenestrated morphology *in vitro* (Géraud et al., 2010; Martinez et al., 2008; Li et al., 2022) freshly isolated cells from mouse liver perfusions were used in all experiments to best reflect *in vivo* cell functions.

We present evidence that dynamin is involved in early MuHV-1 infection in LSECs. We also found that the infection was blocked by monensin, which prevents acidification of endosomes/lysosomes, suggesting uptake of MuHV-1 in LSEC is via endocytosis. Interestingly, infection rates were greatly and significantly inhibited in LSECs when applying a blocking antibody to neuropilin-1; hence this study shows that MuHV-1 binds to neuropilin-1, suggesting that this receptor is important for infection and may serve as a potential entry point for MuHV-1 in mouse LSEC. These observations confirm the recent finding that neuropilin-1 is a receptor for MuHV-1 (Lane et al., 2020).

## Materials and methods

### Animals and ethics

The experiments were done with liver cells from C57Bl/6JRj male mice. The mice were obtained directly from Janvier Lab (France) at the age of 5-6 weeks and acclimatized for at least 5 days, before being included in the experiments. The mice were group-housed (3-4 mice per cage) in cages designed for mice, with aspen bedding, nesting material, houses, and aspen bricks as environmental enrichment (Datesand, Manchester, UK). All mice had free access to fresh water and a standardized mouse diet and were kept under controlled conditions (21°C ± 1°C, relative humidity 55% ± 10%, and 12 h light/12 h dark cycle) at the animal research facility at UiT-The Arctic University of Norway (Tromsø, Norway). In the period before the experiment, the health of the animals was supervised daily by experienced animal technicians.

The entire experimental procedure (liver perfusion for cell isolation) was performed post-mortem in mice that had been euthanized by cervical dislocation. The experimental protocol and animal handling were approved by the competent institutional authority at UiT-The Arctic University of Norway, licensed by the National Animal Research Authority at the Norwegian Food Safety Authority (Approval IDs: UiT 20/21, and 09/22). Experiments were performed in compliance with the European Convention for the Protection of Vertebrate Animals used for Experimental and Other Scientific Purposes.

### Mouse LSEC isolation, purification, and culture

Liver perfusion for cell isolation started immediately after the mice had been euthanized by cervical dislocation. The protocol was as previously described (Elvevold et al., 2022). Briefly, LSECs were purified by immunomagnetic cell separation using endothelium specific CD146 magnetic beads (MACS kit, Miltenyi Biotec, Germany). The cells collected from MACS were spun down and resuspended in RPMI-1640 (Gibco; Thermo Fisher Scientific, Waltham, MA), supplemented only with penicillin (100 IU/ml) and streptomycin (0.1 mg/ml), counted, seeded onto human fibronectin-coated tissue culture plates, and incubated at 37°C in 5% O_2_ and 5% CO_2_ atmosphere. The cultures were gently washed with prewarmed medium 30-40 min post-seeding and then incubated further in RPMI-1640. The total number of LSECs purified from one mouse liver was 4-12 x10^6^ cells. The protocol for CD146 MACS purification of LSECs from liver non-parenchymal cells resulted in highly pure LSEC cultures with > 95% endothelial cells displaying fenestrae (assessed by scanning electron microscopy), which is the morphological hallmark of LSECs (Braet and Wisse, 2002). Freshly isolated LSECs, kept for up to 24 h in cultures, were used in all experiments.

### Dynamin inhibitors

Dynasore (Sigma-Aldrich, Cat. No D7693-5MG) was dissolved in dimethyl sulfoxide (DMSO) according to the manufacturer's protocol and the 31.03 mM stock concentration was stored at -20°C. Working dilutions at concentrations of 0, 40, 80 and 160 µM were prepared freshly on the day of experiment by resuspending stock solution in protein-free medium (RPMI-1640). The same concentration of DMSO as in the 160 µM Dynasore working solution was added to parallel control wells as vehicle control to discriminate DMSO-effects on LSECs morphology, viability, toxicity, and endocytosis functions. Dynasore doses (40, 80, 160 µM) used in this study were determined on the basis of information published by (Macia et al., 2006; Abban et al., 2008; Kirchhausen et al., 2008; Mousavi et al., 2019).

MitMAB (Sigma-Aldrich, Cat. No 32441-500MG) was dissolved in sterile water and stored in stock solution (100 mM) at -20 °C. Working dilutions at concentrations of 2.5 and 5 µM were prepared freshly on the day of experiment by resuspending stock solution in protein-free medium (RPMI-1640).

### Lactate dehydrogenase cytotoxicity assay

Release of LDH to medium was analyzed with the Promega LDH Glo Cytotoxic assay (Cat. No J2380). LSECs (0.5 x 10^6^ cells/well) in fibronectin-coated 24-well tissue culture plates (Sarstedt) were maintained in 0.5 ml RPMI-1640 medium with 0, 40, 80, or 160 μM dynasore, or 0.25% DMSO only (vehicle control). After incubation for 2.5 h and 24 h, cell supernatant (25 μl) was collected and frozen at -20°C in 475 µl LDH storage buffer, until analysis. Parallel cultures were dissolved in Triton X-100 (final concentration 0.1%) and used as maximum LDH release control. Luminescence in the medium was detected at emission 540-550 nm. The experiments were performed with four biological replicates, each done in duplicate.

### Live/dead imaging assay

LSECs (0.2 x 10^6^ cells/well), were seeded in fibronectin-coated 48-well plates (Sarstedt), incubated for 40 min, then washed and incubated further in RPMI-1640 medium with 0, 40, 80, or 160 µM dynasore, or 0.25% DMSO only (vehicle control) for 2.5 h. Cell viability was assessed with Invitrogen LIVE/DEAD™ Cell Imaging Kit (488/570, Cat. No R37601). The reagent was added to be present during the last 15 min of the incubation and cells were imaged in a widefield microscope (Zeiss Cell Discoverer 7). Ten images were taken automatically with 10 x magnification at preset locations within each well, and the live (green) and dead (red) cells were separated and counted automatically with the software Cell Profiler (copyright Broad Institute, Cambridge, Massachusetts). The experiment was repeated with three biological replicates, each done in duplicate.

### Assessment of LSEC morphology by scanning electron microscopy

LSECs (0.5 x 10^6^ cells/well, producing a confluent monolayer culture) were seeded in fibronectin-coated 24-well plates (Sarstedt), incubated for 40 min in RPMI-1640 alone, then in RPMI-1640 with dynasore (0, 40, 80, or 160 µM) or 0.25% DMSO only (vehicle control) for 1.5, 2.5, or 1.5 h followed by 11 h incubation in medium alone. In some experiments, the effect of dynasore in cultures with half of this cell density was also examined. Confluent LSEC cultures were treated with MitMAB (0, 2.5 or 5 µM) for 30 min, 1.5 h, or 1.5 h followed by 11 h incubation in medium alone. All cells were fixed in cold McDowell's fixative for electron microscopy (McDowell and Trump, 1976), and prepared as described (Bhandari et al., 2020). In short, the cultures were washed 3x in PHEM buffer, pH 7, incubated for 1 h in 1% tannic acid in PHEM buffer, washed 3x in PHEM, incubated 1h in OsO_4_ in H_2_O, and dehydrated in 30-100% ethanol before chemical drying in hexamethyldisilazane (Sigma-Aldrich, Merck Life Science, Norway). The specimens were mounted on aluminum stubs, sputter-coated with gold/palladium alloy and scanned in a Zeiss Sigma, or Zeiss Gemini Field Emission Scanning electron microscope (Carl Zeiss, Oberkochen, Germany), both microscopes run at 2kV. At each time point and treatment, high-resolution overview images were taken at random from at least 3 areas per cell culture, and higher magnification images were taken within these areas for detailed analysis. Experiments were repeated with at least three biological replicates.

### Actin stain

LSEC cultures (0.2 x 10^6^ cells/well) established on fibronectin-coated IBIDI 8 well plates (Ibidi) were incubated for 1.5 h with 200 ml RPMI-1640 with 80 µM dynasore, or 0.25% DMSO alone at 37°C. Cells were fixed in 4% buffered formaldehyde for 20 min, then stained for 30 min with Alexa Fluor™ 555 Phalloidin (ThermoFisher Scientific, Cat. No A34055, diluted 1:100 in PBS. Nuclei were stained with DAPI, and samples imaged in a Zeiss LSM 800 confocal laser scanning microscope, using a 40x water objective with NA 1.2.

### Endocytosis of ^125^I-labeled ligands for LSEC scavenger receptors

#### Ligands and labeling

Ribonuclease B, which binds to the mannose receptor (CD206, SR-E3) (Napper and Taylor, 2004) in LSECs was from Sigma Aldrich. Formaldehyde-treated bovine serum albumin (FSA), which binds to several scavenger receptors in LSECs (Blomhoff et al., 1984; Sørensen et al., 2015) was prepared as described (Eskild and Berg, 1984). The ligands were labeled with carrier-free Na^125^I, using Iodogen as oxidizing agent as described by the manufacturer (Pierce Chemicals, Rockford, IL), and separated from unbound ^125^I on a PD-10 column (GE Health, Uppsala, Sweden). The resulting specific radioactivity for both ligands was approximately 1-2 x 10^6^ counts per minute per µg protein.

#### Endocytosis assays

LSEC cultures (0.2 x 10^6^ cells/well) were established in 48-well plates (Sarstedt). To reduce background binding of ligand to substrate, the cell cultures were first incubated for 5 min with 0.1% human serum albumin (HSA) in RPMI-1640. The culture medium was then changed to protein-free RPMI-1640 before treatment of cells with dynasore, because dynasore binds to proteins which reduce the effect of the drug (McCluskey et al., 2013). Cultures were first treated for 30 min at 37°C with dynasore (0, 40, 80, or 160 µM) or 0.25% DMSO only (vehicle control), then ~10 ng ^125^I-labeled ligand was added to each well to a final concentration of 0.1 µg/ml, and cultures incubated further for 2 h with ligand +/- inhibitor.

At the end of the endocytosis experiments radioactivity was analyzed in cells and supernatants, separately, as described (Hansen et al., 2002). Cells were lysed in 1% sodium dodecyl sulphate. Each supernatant was divided into two fractions, one fraction containing intact protein, and the other containing acid-soluble radioactivity. In short, intact protein in the supernatant, containing the non-endocytosed ^125^I-labeled ligand, along with one wash of PBS, and 10 μl of 1% HSA was pelleted with 20% trichloroacetic acid. After adjustment for the percentage of free ^125^I in cell-free control wells, the non-pelleted (acid-soluble) radioactivity in supernatants was taken to represent radioactivity released from cells to the supernatant after intracellular degradation of the endocytosed ^125^I-labeled ligand (Hellevik et al., 1996). Endocytosis was calculated as the sum of cell-associated and acid-soluble radioactivity. Each experiment was repeated with 3-6 biological replicates, each done in duplicate or triplicate. Radioactivity was measured in a gamma counter (Cobra II, Packard).

Parallel LSEC cultures (from the same mouse liver cell preparation) with similar cell density as used in the endocytosis assay were imaged in the widefield microscope Zeiss Cell Discoverer 7 with the live/dead imaging kit to assess viability and cell numbers per culture.

### Endocytosis of FITC-FSA

LSEC cultures (0.2 x 10^6^ cells/well) established on fibronectin-coated IBIDI 8 well plates were preincubated for 30 min at 37°C with dynasore (0, 40, 80, or 160 µM), 0.25% DMSO only (dynasore vehicle control), or MitMAB (0, 2.5, or 5 µM) in 200 μl RPMI-1640. Then, 10 µl fluorescein isothiocyanate labelled FSA (FITC-FSA, stock concentration: 2 mg/ml) was added to each culture to produce a final concentration of 10 µg FITC-FSA per ml medium. Cells were incubated with FITC-FSA for 30 min at 37°C in the continued presence of inhibitor, then the cultures were washed twice in prewarmed medium +/- inhibitor to remove the ligand, and the cells incubated further for 1 h +/- inhibitor. Cells were fixed in 4% formaldehyde in PHEM buffer and nuclei stained with DAPI before being imaged in a Zeiss LSM 800 confocal microscope equipped with a 40x water objective (NA 1.2). All experiments were repeated with at least three biological replicates.

### Production and purification of muromegalovirus muridbeta1 (MuHV-1)

MuHV-1 (ATCC, VR-1399, Smith MSGV strain) were propagated in mouse embryonic fibroblast cells (SC-1, ATCC, CRL-1404) according to previously published protocols for herpesvirus propagation (Britt, 2010; Brizić et al., 2018). In short, virus-inoculated cell culture supernatant was collected 5-7 days after inoculation and centrifuged for 20 min at 4,000 x g to remove cell debris and then filtered through 0.45 μm Filtropur S filters (Sarstedt AG & Co, Nümbrecht, Germany). Viral suspensions were then purified by ultracentrifugation through a sucrose cushion (2.5 ml sterile filtered 20% sucrose (w/v) in Tris-buffered saline, (TN; 0.05 M Tris, 0.1 M NaCl, pH 7.4) in open-top thin wall polypropylene ultracentrifuge tubes (Beckman Coulter, Cat. No 326823). The tubes were centrifuged using an Optima L-90K ultracentrifuge (Beckman Coulter) in a SW28 swinging-bucket rotor at 23,000 rpm (~70,000 x g) for 1 h. The viral pellet was resuspended in 1 to 2 ml TN buffer or PBS, aliquoted in cryotubes and stored at -80°C.

Virus quantification and virion integrity assessments: Aliquots of approximately 50 μl MuHV-1 were pulled from storage at least 24 h after freezing and assessed for virus titer and infectivity by fluorescent forming unit (FFU) determination, and virion integrity by transmission electron microscopy.

### Determination of MuHV-1 fluorescent forming unit

To determine FFU, 1.5 x 10^4^ SC-1 cells/well were seeded in 96-well cell culture plates (Becton Dickinson Labware, Cat. No 353072) in 100 μl complete medium (Eagle’s minimal essential medium (EMEM), 5% FBS, 100 IU/ml penicillin and 0.1 mg/ml streptomycin). Ten-fold serial dilutions (10^-1^ to 10^-6^) of the purified virus stocks in EMEM, 2% FBS, were prepared and added to at least duplicate wells. After incubation for 2 h at 37°C with 5% CO_2_, the virus suspensions were removed, and the wells gently washed twice with medium. 100 μl complete medium was added, and the plates were incubated for another 24 h. The cells were fixed and permeabilized in cold absolute ethanol (VWR, 20821.296) for 20 min at room temperature (RT). Fixed cells were kept in PHEM-buffer until further processing. Staining of virus-infected cells was performed using antibodies targeting MuHV-1 immediate-early 1 antigen (IE1, pp89, m123) expression. The MuHV-1 infected cells were labelled for IE1 expression using the mouse anti-m123/IE1 antibody (Capri, HR-MCMV-12, 2.0 µg/ml). Secondary antibody was Dylight-488 (donkey anti-mouse, Invitrogen Cat. No SA5-10166, 2.5 µg/ml). Zeiss Cell Discoverer 7 was used to take 10 images (image size 31.07 x 41.51 mm) with 20x objective of each cell culture at preset locations for counting of IE1 positive nuclei. Nuclei were stained with DAPI. Average number of cell nuclei per image was 279.

### Transmission electron microscopy

Viral suspension was diluted 1:2 in 8% formaldehyde (Sigma-Aldrich, Cat. No 158127) in PHEM and processed for negative staining and TEM. In short, 400 mesh formvar coated copper grids were glow discharged, and placed on top of 5 μl drops of the viral suspension at RT for 5 min in a moist chamber and subsequently washed in ddH_2_O. Grids were placed on droplets containing 1% uranyl acetate (UA) in ddH_2_O at RT for 20 s, then air-dried. Stained grids were examined with a Jeol JEM 1010 transmission electron microscope (Jeol USA Inc., Peabody, MA, USA) connected to a Morada Camera system (Olympus Soft Imaging Solutions, Münster, Germany) to evaluate the density and integrity of fully enveloped viral particles in the suspensions.

### Use of dynamin-inhibitors to block MuHV-1 infection in mouse LSECs

LSECs (0.25 x 10^6^ cells/well) in fibronectin-coated 48-well tissue culture plates were kept in RPMI-1640 and pretreated for 30 min with dynasore (40, or 80 µM) or MitMAB (2.5, or 5 µM). Different batches of viruses were used in the experiments with dynasore and MitMAB. Controls included were non-treated for MitMAB, and 0.25% DMSO as vehicle control for dynasore. MuHV-1 with a multiplicity of infection (MOI) of 0.2 was added to cell cultures +/- inhibitor and incubated at 37°C for 1 h. Cells were then gently washed and incubated further for 11 h in cell medium without virus or inhibitor. After this period, cells were fixed in absolute ethanol, stained with DAPI and immunolabelled for IE1 expression. The 11 h chase period after the initial 1 h inoculation was based on a pilot experiment testing IE1 expression in LSEC in culture at different time points of MuHV-1 incubation. In this pilot experiment, continuous incubation of LSECs with virus (MOI 0.2) showed 27%, 35%, and 38% IE1 positive cells after 8, 12, and 24 h (data not shown).

The MuHV-1 infected cells were labelled for IE1 expression using the mouse anti-m123/IE1 antibody (Capri, HR-MCMV-12, clone IE1.01, at 0.2 µg/ml). Secondary antibody was Dylight-488 (donkey anti-mouse, Invitrogen Cat. No SA5-10166, at 0.25 µg/ml). DAPI was used as general nuclear stain. Infection rate was measured as the percentage of IE1-positive cells in LSEC cultures and compared to infection rate in control cultures not exposed to dynasore, or MitMAB. We included a control without virus as a negative control. Images were recorded with Zeiss Cell Discoverer 7 at preset locations in the culture wells. In the experiments with dynasore, 8 images were recorded from each culture with 10x objective; average number of nuclei per image were 1466. In the experiments with MitMAB, 10 images were recorded of each cell culture with 20x objective; average number of nuclei per image were 189. Each experiment was performed with at least three biological replicates, each done in duplicate wells.

#### The use of acidification inhibitor monensin on MuHV-1 infection in LSECs

LSEC cultures (0.2 x 10^6^ cells/well) established on fibronectin coated IBIDI 8 well plates were preincubated for 30 min at 37°C with monensin (0, 2 or 4 µM, Cat. No 00-4505-51, ThermoFisher), in 200 μl RPMI-1640. Cultures were incubated with MuHV-1 at a multiplicity of infection (MOI) of 0.2 and in the presence or absence of monensin for 1 h, at 37°C, then gently washed in prewarmed medium and further incubated for 6.5 h without virus or monensin. The cells were fixed in absolute ethanol and immunolabelled for IE1 expression as described before. Images were recorded with Zeiss Cell Discoverer 7 at preset locations in the culture wells. Ten images were recorded from each culture with 20x objective and IE1 positive cells and DAPI stained nuclei were counted in Fiji software. Average number of nuclei per image was 211. The experiments were repeated with three biological replicates, each done in duplicate wells.

#### Immunostaining of LSECs to neuropilin-1

LSEC cultures (0.2 x 10^6^ cells/well) were established on fibronectin-coated IBIDI 8 well plates in RPMI-1640 and incubated for 2 h. The cells were then fixed for 20 min with 4% formaldehyde in PHEM buffer, permeabilized with 0.1% triton X-100 for 4 min, incubated with blocking buffer (1% BSA, 3% donkey serum in PHEM buffer) for 30 min, and labeled with polyclonal goat anti-mouse/rat neuropilin-1 antibody (Cat. No AF566, R&D) at 5 µg/ml, or goat IgG at 5 µg/ml (Cat. No AB-108-C, R&D; negative control) for 1 h. Cells were then washed and treated for 30 min with donkey anti-goat IgG (H+L) cross-adsorbed secondary antibody (5 µg/ml), Alexa Fluor-568 (Cat. No A11057, Invitrogen). Nuclei were stained blue with DAPI. Antibodies were diluted in blocking buffer, and the entire procedure was performed at RT. Images were recorded with a Zeiss LSM800 confocal microscope equipped with a 40x water objective (NA 1.2).

#### Western blot

Mouse LSECs were solubilized in RIPA buffer (Thermo Scientific, Cat. No 89900) containing protease inhibitor cocktail (Roche, Cat. No 04693159001), vanadate, pepstatin A and N-ethylmaleimide, and protein concentration was measured by Direct Detect Spectrometer (Millipore). Samples were sonicated, reduced, and heated at 70°C for 10 min and loaded onto SDS-PAGE NuPage 4-12% Bis-Tris gel (Invitrogen) together with the Precision Plus protein Dual color standard (Cat. No 1610374, Bio-Rad) and the MagicMark western protein standard (Cat. No LC5603, Invitrogen). Immunoblotting was performed on 0.45 µm PVDF transfer membrane (Cat. No 88518, Thermo Scientific). Unspecific signal was blocked by incubation with 1xTBS with 0,1% Tween 20 and 5% low-fat powder milk (blocking buffer) for 1 h at RT, followed by incubation with primary antibody overnight at 4°C (goat anti-mouse/rat neuropilin-1 (Cat. No AF566, R&D; 0.2 µg per/ml). Beta-actin staining was used as loading control (Cat. No ab8227, Abcam). Secondary antibody was donkey anti-goat HRP, Cat. No A16005, Invitrogen; diluted 1:10.000; incubation: 1 h at RT). The stained proteins were visualized with Super SignalWest Pico Plus chemiluminescent substrate (Cat. No 34580, Thermo Scientific) and imaged in ImageQuant LAS4000.

#### Competitive inhibition experiments with neuropilin-1 antibody

LSEC cultures (0.2 x 10^6^ cells/well) were established on fibronectin-coated IBIDI 8 well plates in RPMI-1640 with 1% human serum albumin. LSECs were pretreated with goat anti-mouse/rat neuropilin-1 antibody (Cat. No AF566, R&D; 10 µg/ml), or control IgG (goat IgG, R&D Cat. No AB-108-C; 10 µg/ml), at 4°C for 30 min. Then, MuHV-1 with a MOI of 0.2 was added to cell cultures +/- antibody and cells kept for another 1 h at 4°C to allow virus binding to cells. The cultures were then gently washed with cold RPMI-1640, moved to 37°C and incubated further for 8 h in cell medium without virus and antibody. After this period, the cells were fixed in absolute ethanol, stained and immunolabelled for IE1 expression as described before. Ten images were taken automatically with Zeiss Cell Discoverer 7 at 20 x magnification at preset locations within each well, and the nuclei were counted in Fiji software. Average number of nuclei per image was 135. This experiment was performed with 4 biological replicates, each done in duplicate wells.

### Statistical analyses and presentation of data

Data analyses were performed with SPSS 29.0 software (IBM, Chicago, IL, USA). One-way analysis of variance (ANOVA) was followed by Tukey HSD *post hoc* tests. Repeated measures ANOVA and Mann Whitney U test was used in analyses of LDH assay data. Differences were considered significant if *p* < 0.05. Figures were prepared with Microsoft Excel and Adobe Illustrator software.

## Results

To establish the protocol for the use of dynasore and MitMAB in endocytosis experiments in primary LSEC cultures, we evaluated the dose- and time dependent effects of the drugs with multiple assays to reveal effects on cell morphology, viability, and endocytic function. Results for the two inhibitors are presented separately.

### Effects of dynasore on LSEC viability and morphology

**Figure 1:**
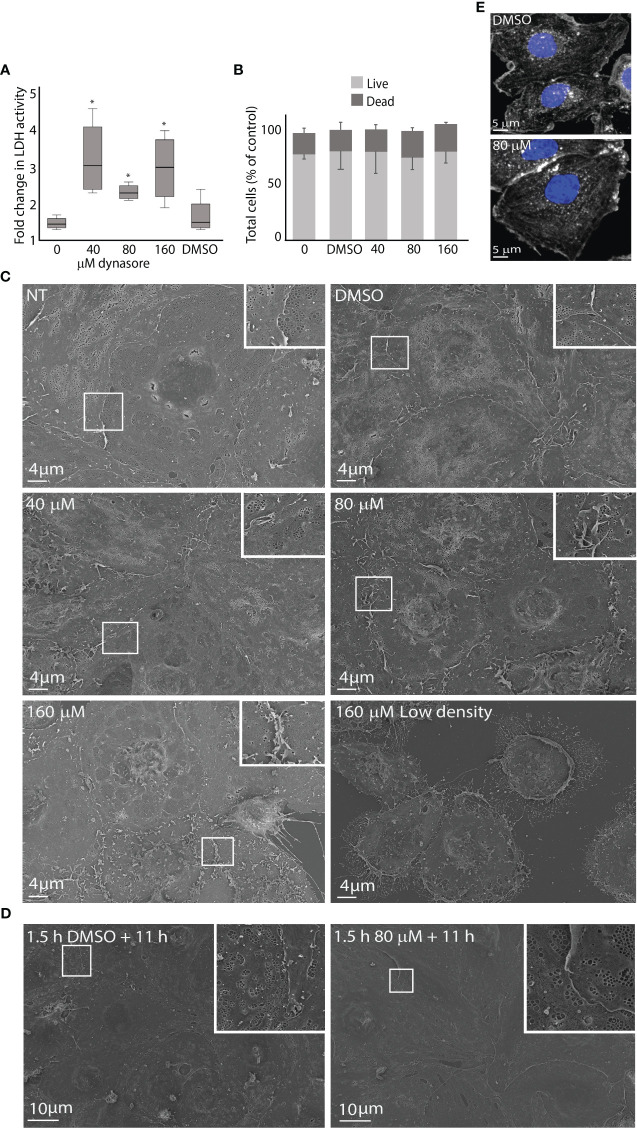
Effects of dynasore on mouse LSEC viability and morphology in culture. **(A)** The figure shows the increase in lactate dehydrogenase (LDH) activity in culture medium from 2.5 to 24 h after treatment of LSEC cultures with 0, 40, 80 or 160 µM dynasore or 0.25% DMSO (vehicle control). The box graph presents the median fold change and upper and lower interquartile range for each group. *Significant different from non-treated control (p-value < 0.05; n=4; Mann Whitney U test). The raw data are presented in **Supplemental Figure 1**: At 2.5 h the LDH levels were not significantly different between groups (One-way ANOVA). **(B)** Bar chart illustrating the ratio between live and dead cells in primary LSEC cultures analyzed with a live/dead imaging kit. LSECs were treated for 2.5 h with dynasore (40, 80, 160 µM), or 0.25% DMSO only (vehicle control). Results are normalized to non-treated control (0). Results are averages ± SD of 3 biological replicates. Statistical analysis (One-way ANOVA) showed no significant difference between groups. **(C)** Scanning electron micrographs of freshly isolated LSECs seeded in confluent monolayers (normal density) treated with 40, 80 or 160 µM dynasore, or 0.25% DMSO only for 2.5 h. (NT=not treated control). Lower lane, right image: LSEC culture seeded with low cell density (half of normal) and treated with 160 µM dynasore for 2.5 h. **(D)** LSEC cultures (normal density) treated for 1.5 h with 80 µM dynasore or 0.25% DMSO only and incubated further for 11 h in medium without treatment. **(E)** Effect of dynasore on the actin cytoskeleton in LSEC. The cells were treated for 1.5 h with 0.25% DMSO or 80 µM dynasore. Actin filaments were stained with phalloidin-550 (white) and cell nuclei with DAPI (blue).

Cytotoxic effect of dynasore on LSECs leading to loss of plasma membrane integrity was monitored with an LDH release assay. The LDH levels in cell supernatants were measured at 2.5 and 24 h. After 2.5 h no significant differences in LDH levels were observed between the dynasore-treated groups and control groups, while after 24 h, LDH increased more in supernatants in dynasore-treated groups than in the control groups (**Figure 1A**; **Supplemental Figure 1**). Phase contrast microscopy (not shown) revealed cell detachment and shrinkage of attached cells with dynasore after 24 h. We thus decided to use shorter treatments in further experiments.

Viability of LSECs was analyzed with a live/dead imaging assay after dynasore exposure for 2.5 h. This experiment did not reveal significant differences in total number of attached cells, nor in the ratio of live and dead cells between dynasore-treated cultures, and non-treated or vehicle control cultures (**Figure 1B**).

To examine dose- and time-dependent effects of dynasore on LSEC ultrastructure, cell cultures were examined by scanning EM after exposure to dynasore (40, 80 or 160 µM) for 2.5 h (same conditions as used in endocytosis experiments with ^125^I-labeled ligands), or 1.5 h followed by 11 h in medium alone (same conditions as used in virus infection experiments). For each time point, cultures from at least 3 individual mice were examined. Typical observations are shown in **Figure 1C**. LSECs exposed to 40 µM dynasore for 2.5 h resembled control cultures (**Figure 1C**). The cells were well fenestrated with fenestrae organized in sieve plates, which is the typical morphological feature of LSECs (Wisse, 1970; Szafranska et al., 2021). Increased membrane ruffles were evident in LSECs exposed to 80 µM dynasore compared to control cultures but the cells were still well fenestrated. Cells exposed to 160 µM dynasore for 2.5 h had less fenestrations and increased membrane ruffling compared to lower doses of the drug. Notably, when we compared LSECs in confluent monolayer cultures with LSECs seeded to produce half-confluent cultures, the latter showed marked cell shrinkage and cellular detachment after exposure to 160 µM dynasore for 2.5 h (**Figure 1C**, lower row, right image). This suggests that dynasore is more toxic at low LSEC density in culture. We therefore used dense monolayer cultures in all experiments with dynasore and MitMAB.

Dynasore exposure (40-80 µM) for 1.5 h followed by 11 h in medium alone did not change the LSEC morphology compared to control as revealed by scanning EM (**Figure 1D**).

Dynasore has been reported to destabilize the actin cytoskeleton (Yamada et al., 2009; Wang Q.-C. et al., 2015). We therefore treated LSECs for 1.5 h with 80 µM dynasore (the highest dose used in virus infection experiments) and labeled actin filaments with F-actin probe Alexa Fluor 555™ phalloidin. This did not reveal visible changes in the overall organization of the actin cytoskeleton (**Figure 1E**).

### Dynasore significantly inhibited endocytosis of scavenger and mannose receptor ligands in LSEC

**Figure 2:**
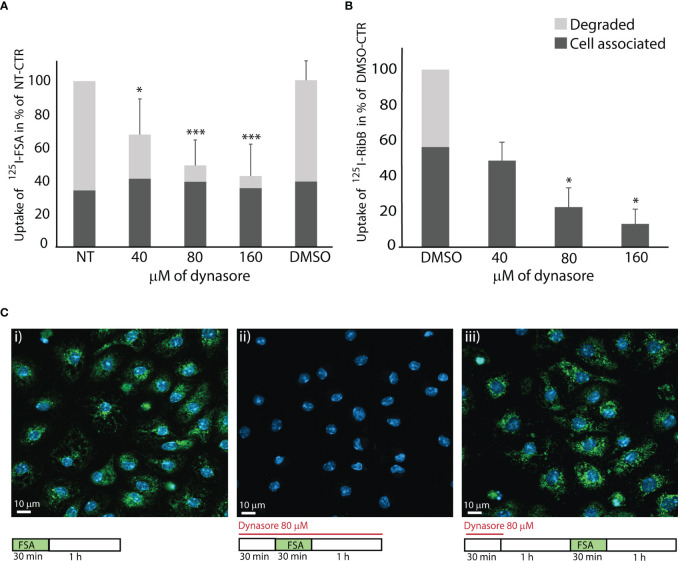
Dynasore inhibits endocytosis in LSECs of ligands for scavenger and mannose receptors. The bar charts in A and B illustrate the inhibitory effect of dynasore on LSECs endocytosis of **(A)** ^125^I-FSA (formaldehyde-treated serum albumin) and **(B)** ^125^I-RibB (Ribonuclease B). Cells were pretreated for 30 min with dynasore (40, 80, or 160 µM) before the ligand (^125^I-FSA or ^125^I-RibB) was added. Cells were then incubated with ^125^I-FSA or ^125^I-RibB in presence of 40, 80, or 160 µM dynasore, or 0.25% DMSO for 2 h at 37 ˚C. Cell-associated and degraded ligand were measured as described in Materials and Method, and results are given in percent of non-treated control (+/- SD). Biological replicates: ^125^I-FSA, n=6; ^125^I-RibB, n=3 except for 40 µM dynasore, where n=2. *p < 0.05; ***p < 0.001 (One-way ANOVA). Images in **(C)** shows effects of dynasore on endocytosis of FITC-FSA (green) in LSEC cultures (representative images of 3 biological replicates): i. Control. LSECs in cell medium with 0.25% DMSO incubated 30 min with FITC-FSA (green) followed by a 1 h chase without ligand. ii. LSECs pretreated for 30 min with 80 µM dynasore before 30 min incubation with FITC-FSA and dynasore, followed by a 1 h chase (without ligand) in the presence of dynasore iii. Recovery of LSEC endocytosis after 30 min treatment with 80 µM dynasore, followed by 1 h recovery time in cell medium before 30 min incubation with FITC-FSA. Nuclei stained with DAPI (blue), and images were captured with a Zeiss LSM 800 confocal laser scanning microscope.

In the next set of experiments, we examined the effect of dynasore on LSEC endocytosis. We first tested the uptake of two ^125^I-labeled proteins for LSEC receptors that operate via clathrin-mediated endocytosis (Magnusson and Berg, 1989; Magnússon and Berg, 1993; Hansen et al., 2005). The ligands were FSA which binds to stabilin-1 and stabilin-2 (McCourt et al., 1999; Li et al., 2011), and ribonuclease B which binds to the mannose receptor (Napper and Taylor, 2004). Endocytosis of ^125^I-FSA is presented in **Figure 2A**. Total endocytosis, i.e. the sum of cell-associated and degraded ^125^I-FSA was approximately 40% (± 11%, n=6) of added ligand in control cultures after 2 h. Dynasore significantly inhibited degradation of ^125^I-FSA in a dose-dependent manner, indicating that the internalization of ligand was reduced or blocked, while there was a fraction of ligand associated with the cells that were not inhibited by dynasore. Degradation of ^125^I-FSA was almost completely blocked with 80 and 160 µM dynasore.

Uptake of ^125^I-ribonuclease B in LSEC cultures was approximately 8% (± 3.5%, n=3) of added ligand after 2 h incubation. Dynasore significantly reduced cell-associated ligand radioactivity, and blocked ligand degradation in LSEC cultures (**Figure 2B**).

FITC-labeled FSA was used to visualize ligand uptake in LSECs. We found that FITC-FSA was endocytosed by all cells in vehicle-treated control cultures, while there was no visible uptake in cells treated with dynasore (80 µM) (**Figure 2C**). To examine if LSECs can regain their endocytosis function after inhibitor treatment we exposed the cells to dynasore for 30 min followed by 1 h in medium without inhibitor to allow the cells to recover their endocytosis function, before FITC-FSA was added to the cultures. This experiment showed efficient uptake after the recovery period (**Figure 2C**).

### Effects of MitMAB on LSEC morphology and endocytosis

**Figure 3:**
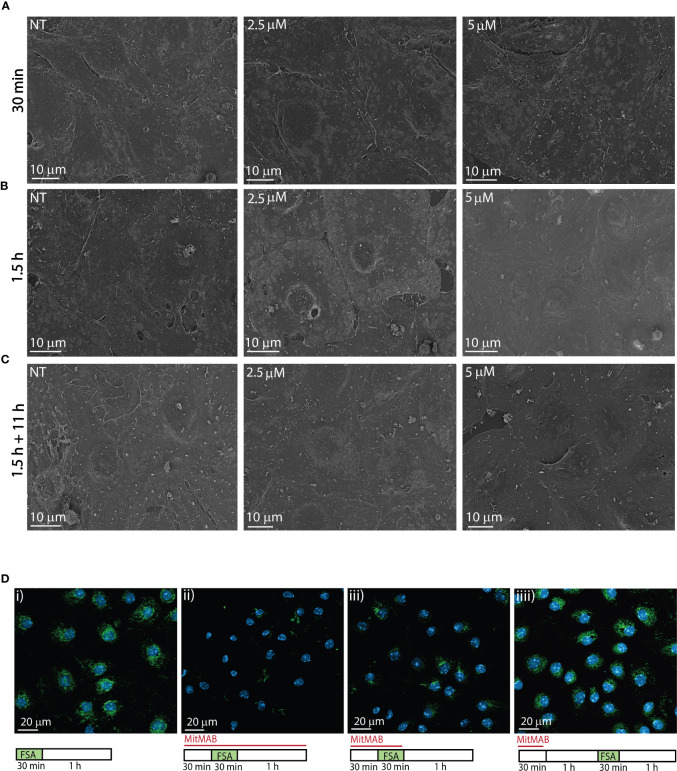
Effects of MitMAB on LSEC morphology and endocytosis. **(A–C)** Scanning electron micrographs shows freshly isolated LSECs seeded in confluent monolayers (normal density) treated with 2.5 or 5 µM MitMAB for **(A)** 30** **min, **(B)** 1.5 h or **(C)** 1.5 h followed by 11 h without MitMAB. (NT=non treated control). Images are representative for the results in 3 biological replicates. **(D)** show the effect of MitMAB on LSEC endocytosis of FITC-FSA (green). N=3. i. Control. LSECs in cell medium (RPMI-1640) incubated 30 min with FITC-FSA (green) followed by a 1 h chase without ligand. ii. LSECs pretreated for 30 min with 5 µM MitMAB before 30 min incubation with FITC-FSA and MitMAB, followed by a 1 h chase (without ligand) in the presence of MitMAB. iii. LSECs pretreated for 30 min with 5 µM MitMAB before 30 min incubation with FITC-FSA and MitMAB, followed by a 1 h chase without inhibitor. iv. Recovery of LSEC endocytosis after 30 min treatment with 5 µM MitMAB, followed by 1 h recovery time in cell medium before 30 minutes incubation with FITC-FSA. Nuclei stained with DAPI (blue), and images were captured with a Zeiss LSM 800 confocal laser scanning microscope.

The morphology of LSECs in cultures treated for 30 min with MitMAB (2.5 or 5 µM) appeared similar to the cell morphology of control cultures when evaluated by scanning EM (**Figure 3A**). After 1.5 h exposure, cells exposed to 5 µM MitMAB showed smoother, and more tightly sealed cell borders with few gaps between cells, compared to non-treated cells. We also observed reduced fenestration and less membrane ruffles compared to control cells (**Figure 3B** **).** After 1.5 h exposure to MitMAB (2.5, 5 µM) followed by 11 h in medium, the cells produced confluent monolayers but with reduced formation of sieve plates and smoother cell borders compared to non-treated cultures (**Figure 3C**).

MitMAB effectively inhibited endocytosis of FITC-FSA in LSECs (**Figure 3D**). As observed with dynasore (**Figure 2C**), the cells were able to recover their endocytosis function after 30 min of exposure to MitMAB.

### Dynasore and MitMAB significantly blocked MuHV-1 infection in mouse LSEC

**Figure 4:**
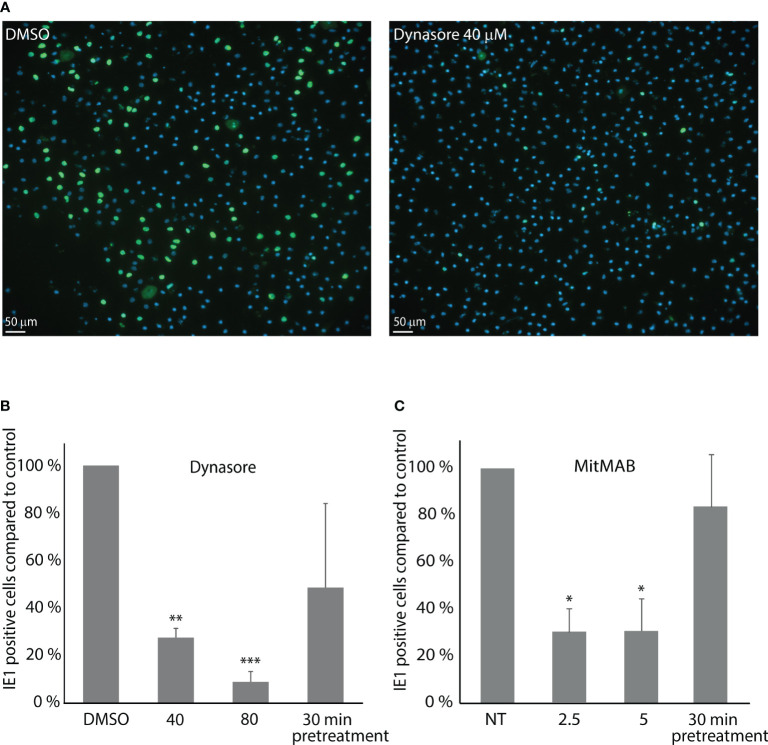
Dynasore and MitMAB inhibit LSEC early infection with muromegalovirus muridbeta1. LSEC cultures pretreated for 30 min with dynasore or MitMAB were incubated with MuHV-1 (MOI 0.2) for 1 h, washed and incubated further for 11 h without virus/inhibitor. Fixed LSECs were labelled with an antibody to IE1 (green). Nuclei stained blue with DAPI. The rate of infected cells was analyzed by quantifying early immediate antigen (IE1)-positive cells (green) as described in Methods. **(A)** Images show markedly reduced number of MuHV-1 infected cells in LSEC cultures treated with 40 µM dynasore compared to DMSO-treated control cells. **(B, C)** Bar charts illustrating the ratio of MuHV-1 infected (IE1-positive) cells in cultures with **(B)** dynasore, or **(C)** MitMAB. The results in each figure are averages of 3 biological replicates ± SD and infection in non-treated control LSEC cultures are set as 100%. *p < 0.05; **p < 0.01; ***p<0.001 (One-way ANOVA).

MuHV-1 infection in primary mouse LSECs was examined by counting the number of IE1 positive nuclei per culture. Cultures inoculated with a MOI of 0.2 for 1 h followed by 11 h incubation in cell medium only, showed up to 25% IE1 positively stained nuclei in control cultures (**Figure 4**). Averaged infection rate in control cultures were 22.5% (± SD 4.6%) in dynasore experiments and 8.3% (± SD 1.3%) in MitMAB experiments. Notably, different batches of viruses were used for these experiments.

The number of IE1 positive cells (i.e., infected cells) was decreased by approximately 70% and 90% (n=3) in LSEC cultures treated with 40 µM, or 80 µM dynasore, respectively (**Figure 4A**), while the number of MuHV-1 infected cells decreased by around 70% in the experiments with MitMAB (**Figure 4B**). Pretreatment with dynasore, or MitMAB for 30 min followed by virus inoculation for 1 h without inhibitor and 11 h chase, only slightly reduced the infection rate, suggesting that the effect of the inhibitor is reversible.

#### Neuropilin-1 is involved in MuHV-1 infection in mouse LSEC

**Figure 5:**
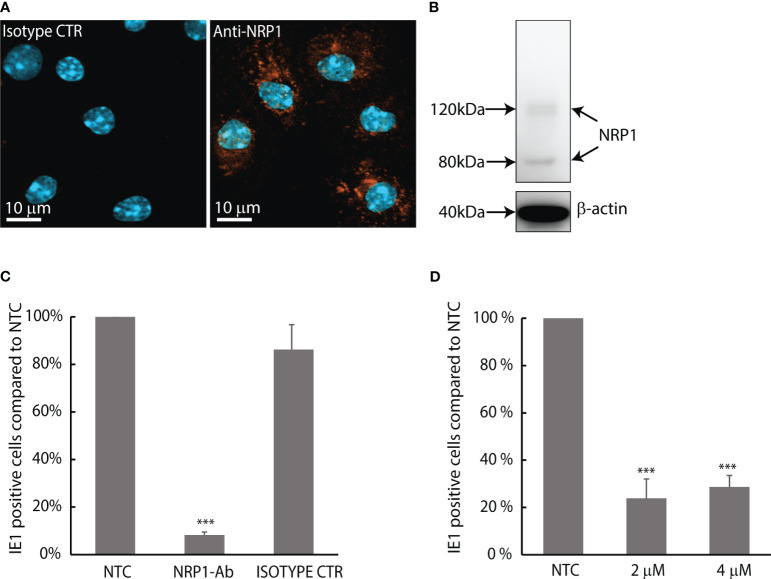
MuHV-1 binds to neuropilin-1; infection in LSEC is pH-dependent. **(A)** Confocal micrograph of mouse LSECs labelled with a polyclonal antibody to mouse/rat neuropilin-1 (NRP1). Positive staining is seen as orange fluorescence. Cell nuclei are stained blue with DAPI. **(B)** Western blot of neuropilin-1 expression in mouse LSECs using the same antibody, β-actin were used as loading control. **(C)** Competitive binding assay with goat anti-mouse/rat neuropilin-1 antibody. LSEC cultures were inoculated with MuHV-1 (MOI 0.2) in the presence or absence of neuropilin-1 antibody or non-immune goat IgG control at 4°C and allowed to bind virus for 1 h; then washed with cold medium and moved to 37°C and incubated for 8 h in medium without virus and antibody. Fixed cells were immunolabelled for IE1 and the rate of infected cells was analyzed by quantifying IE1-positive cells in culture. Results are presented as % of non-treated control. Infection rate of the non-treated control cultures was 3.6% ± 0.6%. Results are averages ± SD of 4 biological replicates. ***p < 0.001 (One-way ANOVA). **(D)** Effect of monensin on the infection rate of MuHV-1 in LSEC. Cells were incubated with MuHV-1 (MOI 0.2) for 1 h ± monensin at 37°C, washed and incubated for another 6.5 h at 37°C omitting virus and monensin. Fixed cells were immunolabelled for IE1. Results are presented as % of non-treated control. Infection rate in control cultures were 9.4% ± 0.4%. Results are averages ± SD of 3 biological replicates. ***p < 0.001 (One-way ANOVA).

MuHV-1 binding to neuropilin-1 is important for infection in endothelial, fibroblast and macrophage cell lines (Lane et al., 2020). To confirm that neuropilin-1 is expressed in mouse LSEC, we did immunostaining of LSEC cultures and western blotting of LSEC protein lysates using a polyclonal antibody to neuropilin-1. Positive staining was observed in all LSECs (**Figure 5A**), and the western blot analysis revealed two bands at approximately 80 and 120 kDa (**Figure 5B**). Previous reports have found neuropilin-1 to be a 120-130 kDa transmembrane glycoprotein (Campos-Mora et al., 2013). To examine whether neuropilin-1 is involved in MuHV-1 infection we measured the effect of the same anti-neuropilin-1 antibody in a competitive binding assay. This was done in cold temperature to let the virus bind to cells but inhibit internalization. Virus incubation in the presence of antibody at 4°C, followed by a chase period of 8 h at 37°C significantly decreased the number of IE1 positive cells by 92% (± 1.3%) compared to non-treated control, and 90% (± 10%) compared to the IgG control (**Figure 5C**).

#### MuHV-1 infection of LSEC was inhibited in the presence of monensin

To further investigate the infection pathway of MuHV-1 in LSEC, we examined whether MuHV-1 infection was pH-dependent by using monensin. Monensin is an ionophore that inhibits acidification of endosomes and has been repeatedly used in LSEC endocytosis studies (Hellevik et al., 1996; Martin-Armas et al., 2006; Øie et al., 2020). The number of IE1 positive cells was significantly reduced by 74% (± 6%) in the presence of monensin (**Figure 5D**), suggesting that the transport of virus is dependent on acidification of endosomes.

## Discussion

LSECs are known to be immune tolerogenic, and *in vivo* studies provided evidence that MuHV-1 establish viral latency within the liver in LSECs (Seckert et al., 2009). However, the route of entry has not been characterized in LSECs, and there are currently few studies that describe the early stages of MuHV-1 infection in mouse cells. Lane et al. (2020) identified neuropilin-1 as an important mediator of early infection and a potential entry receptor for MuHV-1 in the endothelial cell line SVEC4-10, fibroblast cell line 3T3-SA and macrophage cell line IC-2 (Lane et al., 2020). In the endothelial cells, heparan sulphate was also involved in virus binding. The route of entry of MuHV-1 into the cells was not described. In the present study, we show that early infection of primary mouse LSECs with MuHV-1, evidenced as nuclear expression of the viral IE1 antigen, was significantly reduced when infecting cells in the presence of a neuropilin-1 blocking antibody (same antibody as used in the study by (Lane et al., 2020). This suggests that MuHV-1 infection in LSEC is dependent on neuropilin-1.

Neuropilin-1 is a transmembrane receptor important for physiological angiogenesis found on vascular endothelial cells and is also a receptor for the vascular endothelial growth factor (VEGF)-A (Fantin et al., 2013). Neuropilin-1 have been reported as entry point for several viruses (Perez-Miller et al., 2021), including SARS-CoV-2 (Cantuti-Castelvetri et al., 2020), Epstein-Barr virus (Wang H. B. et al., 2015) and human T-lymphotropic virus-1 (Lambert et al., 2009). Neuropilin-1 undergoes endocytosis in response to ligand binding and is known to utilize different pathways of endocytosis, dependent on the ligand: Binding to semaphorin (sema3c) induces lipid raft-dependent endocytosis, while binding to VEGF-A_165_ induces clathrin-dependent endocytosis (Salikhova et al., 2008). Interestingly, endocytosis of VEGF-A in rat LSECs was reported to occur via clathrin-mediated dynamin-dependent endocytosis (Mousavi et al., 2019).

Furthermore, we found that the dynamin-inhibitors dynasore and MitMAB, which function via different mechanisms, markedly and significantly reduced infection rates in mouse LSECs. This suggests that viral entry, and/or transport to the nucleus is dynamin-dependent in these cells. When acidification of endosomes was inhibited by the ionophore monensin, the infection rate was significantly reduced, indicating that the MuHV-1 infection is pH-dependent, strengthening the notion that virus uptake in LSECs is via endocytosis. The cells represent a unique type of fenestrated endothelial cells with high capacity for clathrin-mediated, and thus dynamin-dependent endocytosis (Bhandari et al., 2021). This differs from other endothelial cells where caveolin-mediated endocytosis is a dominating endocytic pathway (Predescu and Palade, 1993). Additional studies are, however, required to address whether the internalization process in LSECs occur via clathrin-coated pits.

Invasion mechanisms used by viruses to infect host cells differ across viral families (Pelkmans and Helenius, 2003), but most viruses infect the cell via endosomal pathways to cause a productive infection (Yamauchi and Helenius, 2013). Since dynasore was discovered by Macia et al. (2006) in a large screening of small molecule inhibitors (Macia et al., 2006), it has been widely used to study mechanisms of endocytosis (Kirchhausen et al., 2008). Dynasore is reported to inhibit the cellular entry of a wide range of viruses, including SARS-CoV-2 (spike protein) (Bayati et al., 2021), bluetongue virus (Gold et al., 2010), African swine fever virus (Hernaez and Alonso, 2010), herpes simplex virus 1 (Rahn et al., 2011), dengue virus (Carro et al., 2018) and Japanese encephalitis virus (Zhu et al., 2012). Additionally, dynasore has been reported to prevent the establishment of the MuHV-1 assembly compartments in the early phase of infection in Balb3T3 fibroblasts (Štimac et al., 2021). Furthermore, investigation of MuHV-1 infection in triple dynamin knock-out cells (mouse embryonic fibroblasts), concluded that dynamin is also important for the completion of MuHV-1 maturation (Hasan et al., 2018). Together with our results in primary mouse LSECs, which showed that small molecule dynamin inhibitors could block initial steps of infection, this suggests that dynamin plays a role in several stages of MuHV-1 infection, including viral entry and/or transport to the nucleus, establishment of assembly compartments, virion production and maturation.

As most other drugs, dynamin inhibitors also have off-target effects. Dynasore influences the amount of cellular cholesterol (Park et al., 2013) and may cause destabilization of F-actin (Preta et al., 2015). Therefore, we stained actin as described in Materials and Methods, and observed that actin organization in dynasore-treated LSEC resembled non-treated control cells. Secretion of apoE and other proteins from primary human macrophages was reduced by both dynasore and MitMAB (Kockx et al., 2014). Dynasore did not affect microtubule stability in this study, while MitMAB were reported to increase the levels of acetylated tubulin (Kockx et al., 2014).

Protocols for the use of dynamin inhibitors with primary LSECs are incompletely described, as is the effects of these drugs in the cells. We therefore investigated the dose- and time-dependent effects of dynasore and MitMAB on viability, morphology, and endocytosis of protein ligands in LSECs to establish a safe protocol for their use in studies of MuHV-1 infection. Notably, LSECs rapidly lose cell specific functions *in vitro* (Géraud et al., 2010; Li et al., 2022), and currently there are no cell lines with a preserved cell typical phenotype (Poisson et al., 2017). Experiments was therefore performed in early primary cultures. As dynamin is crucial for clathrin-mediated endocytosis, and LSECs are professional pinocytic cells operating essentially via this pathway and thus have a very high membrane traffic (Sørensen et al., 2012; Sørensen et al., 2015; Bhandari et al., 2021), the cells are likely to be vulnerable to inhibition of dynamin. By carefully evaluating the toxicity of dynasore with two different bioassays and scanning EM we found that dynasore in doses of 40 and 80 µM was well tolerated by the cells for 2.5 h. These concentrations of dynasore significantly inhibited LSEC degradation of the radiolabelled ligands FSA and ribonuclease B in the quantitative endocytosis assays, and thus uptake via scavenger and mannose receptors that constitutively recycle via clathrin-mediated endocytosis in LSECs (Magnusson and Berg, 1989; Hansen et al., 2005). Experiments with radiolabeled ligands produce information about endocytosis per culture but do not discriminate between uptake in individual cells. We therefore also established an assay where we used FITC-FSA to assess the effect of dynamin inhibitors on individual cells in culture by fluorescence microscopy. This assay was used to visualize inhibition of LSEC endocytosis by both dynasore and MitMAB. The FITC-FSA assay corresponds to the commonly used transferrin assay (Mayle et al., 2012; Hirschmann et al., 2015). However, instead of measuring ligand uptake via the transferrin receptor, the uptake is via scavenger receptors that are highly expressed in LSECs (Bhandari et al., 2021).

While safe to use in the time and doses described above, we observed that after 24 h exposure to dynasore, many LSECs were damaged or dead, which shows that time of exposure is critical for cell viability. This finding contrasts with reports in some cell lines where dynasore was used for up to 48 h (Abban et al., 2008; Mues et al., 2015; Zhong et al., 2019; von Beek et al., 2021). This may be explained by the fact that cell lines are often highly different from the true primary cells. In addition, the use of serum supplementation reported in some studies may have reduced the toxicity of dynasore. Dynasore binds to albumin, which reduces the effect of the drug in cells (Kirchhausen et al., 2008; Preta et al., 2015). In the present study, we therefore used serum-free medium in all experiments. The effect of dynasore is further related to cell density, and the general advice for using this drug with cell lines is to evaluate the effect at a cell density of 40-70% (Kirchhausen et al., 2008). However, primary mouse LSECs do not proliferate *in vitro* and thrive best when seeded in a concentration that produces a confluent monolayer. When exposed to a high concentration of dynasore (160 μM) for 2.5 h, we observed increased cell detachment and cell death in half-confluent cultures, as assessed by scanning EM. These findings underline the importance of carefully testing potential toxic effects of endocytosis inhibitors in the cell system used, as primary cells differ from cell lines in multiple aspects.

## Conclusion

Our study gives new insight about the early stages of MuHV-1 infection in primary mouse LSECs, a relevant target cell known to be an important site of viral latency in the liver. We present evidence that MuHV-1 binds to neuropilin-1 in LSECs, and that the early steps of MuHV-1 infection are dynamin-, and pH-dependent in these cells.

## Data availability statement

The original contributions presented in the study are included in the article/**Supplementary Material.** Further inquiries can be directed to the corresponding author.

## Ethics statement

The animal study was approved by The competent institutional authority at the UiT-The Arctic University of Norway, licensed by the National Animal Research Authority at the Norwegian Food Safety Authority. The study was conducted in accordance with the local legislation and institutional requirements.

## Author contributions

Conceptualization: IK and KS. Methodology: IK, AL, JSR, RL and KS. Investigation: IK, AL, JSR, JS-S, RL and KS. Formal analysis: IK, RL and KS. Visualization: IK. Funding acquisition: KS. Project administration: IK and KS. Writing -original draft: IK and KS. Writing -review & editing: IK, AL, JSR, JS-S, RL and KS. All authors contributed to the article and approved the submitted version.

## Supplementary material

Supplementary Figure 1 Effects of dynasore on LDH release in mouse LSEC culture. The figure shows LDH activity in culture medium after treatment with 0, 40, 80 or 160 µM dynasore or 0.25% DMSO (vehicle control). LDH was measured in the same culture at 2.5 h and 24 h. Parallel control cultures were treated with Triton X-100 (TX-100) at 2.5 and 24 h (maximum LDH release control). Results are average values ± SD of 4 biological replicates. *LDH release was significantly increased from 2.5 to 24 h in all groups (Repeated measures ANOVA, p-value < 0.01) but not significantly different between groups at 2.5 h (One-Way ANOVA). Differences in fold change from 2.5 to 24 h between groups are presented in **Figure 1A**.
