# Supplementary figures and images for: Infection of liver sinusoidal endothelial cells with Muromegalovirus muridbeta1 involves binding to neuropilin-1 and is dynamin-dependent
Source: Front Cell Infect Microbiol. 2023 Nov 9;13:1249894. doi: 10.3389/fcimb.2023.1249894 (PMC10665495; doi:10.3389/fcimb.2023.1249894)

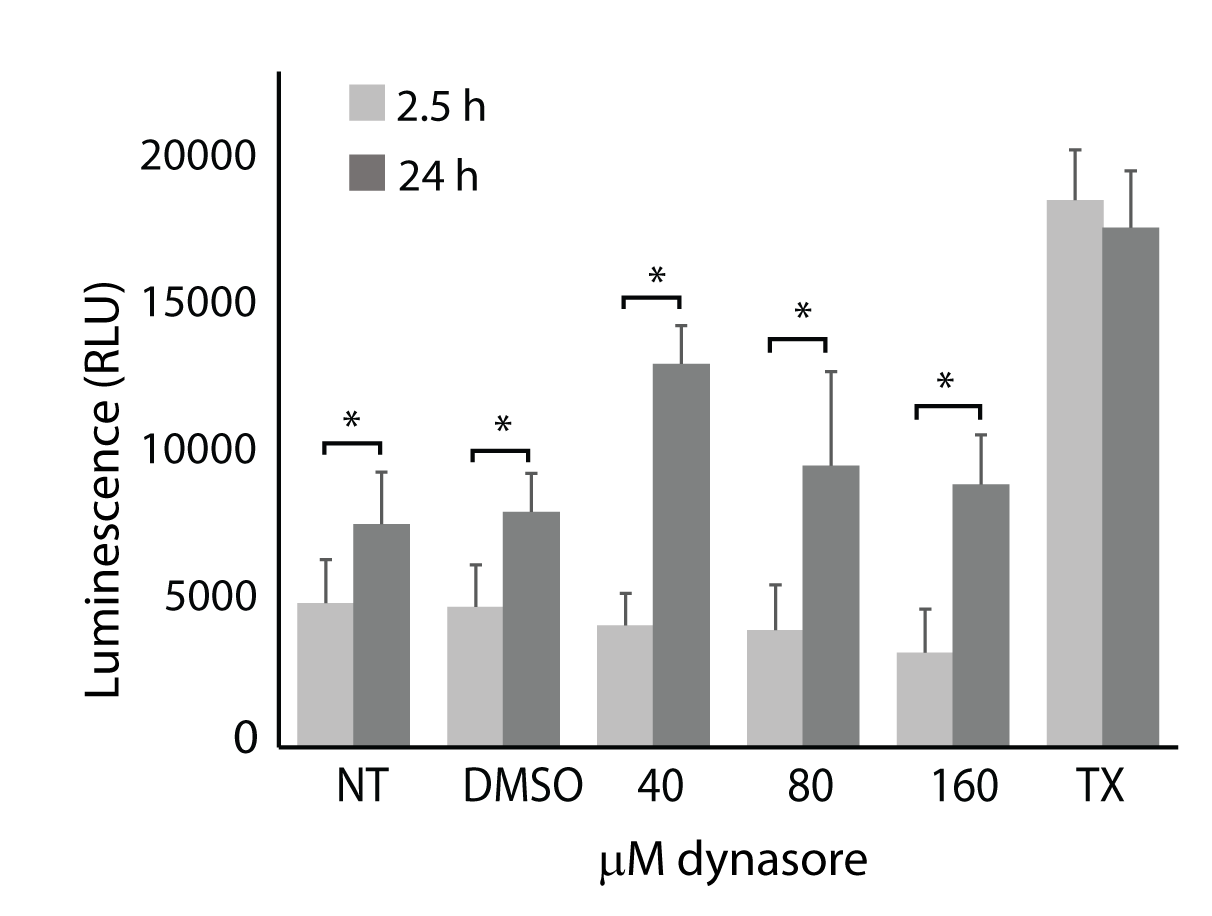

Supplement: Supplementary Figure 1 — Effects of dynasore on LDH release in mouse LSEC culture. The figure shows LDH activity in culture medium after treatment with 0, 40, 80 or 160 µM dynasore or 0.25% DMSO (vehicle control). LDH was measured in the same culture at 2.5 h and 24 h. Parallel control cultures were treated with Triton X-100 (TX-100) at 2.5 and 24 h (maximum LDH release control). Results are average values ± SD of 4 biological replicates. *LDH release was significantly increased from 2.5 to 24 h in all groups (Repeated measures ANOVA, p-value < 0.01) but not significantly different between groups at 2.5 h (One-Way ANOVA). Differences in fold change from 2.5 to 24 h between groups are presented in Figure 1A . [file Image_1.tif]
